# The telomerase inhibitor imetelstat differentially targets JAK2V617F versus CALR mutant myeloproliferative neoplasm cells and inhibits JAK-STAT signaling

**DOI:** 10.3389/fonc.2023.1277453

**Published:** 2023-10-24

**Authors:** Kathrin Olschok, Bianca Altenburg, Marcelo A. S. de Toledo, Angela Maurer, Anne Abels, Fabian Beier, Deniz Gezer, Susanne Isfort, Katrin Paeschke, Tim H. Brümmendorf, Martin Zenke, Nicolas Chatain, Steffen Koschmieder

**Affiliations:** ^1^ Department of Hematology, Oncology, Hemostaseology, and Stem Cell Transplantation, Faculty of Medicine, RWTH Aachen University, Aachen, Germany; ^2^ Center for Integrated Oncology Aachen Bonn Cologne Düsseldorf (CIO ABCD), Aachen, Germany; ^3^ Institute of Clinical Chemistry and Clinical Pharmacology, University Hospital Bonn, Bonn, Germany

**Keywords:** Myeloproliferative neoplasms (MPN), imetelstat (GRN163L), myelofibrosis (MF), induced pluripotent stem cells, telomere length (TL), JAK2V617F, CALR mutations

## Abstract

Imetelstat shows activity in patients with myeloproliferative neoplasms, including primary myelofibrosis (PMF) and essential thrombocythemia. Here, we describe a case of prolonged disease stabilization by imetelstat treatment of a high-risk PMF patient enrolled into the clinical study MYF2001. We confirmed continuous shortening of telomere length (TL) by imetelstat treatment but observed emergence and expansion of a KRAST58I mutated clone during the patient’s clinical course. In order to investigate the molecular mechanisms involved in the imetelstat treatment response, we generated induced pluripotent stem cells (iPSC) from this patient. TL of iPSC-derived hematopoietic stem and progenitor cells, which was increased after reprogramming, was reduced upon imetelstat treatment for 14 days. However, while imetelstat reduced clonogenic growth of the patient’s primary CD34+ cells, clonogenic growth of iPSC-derived CD34+ cells was not affected, suggesting that TL was not critically short in these cells. Also, the propensity of iPSC differentiation toward megakaryocytes and granulocytes was not altered. Using human TF-1^MPL^ and murine 32D^MPL^ cell lines stably expressing JAK2V617F or CALRdel52, imetelstat-induced reduction of viability was significantly more pronounced in CALRdel52 than in JAK2V617F cells. This was associated with an immediate downregulation of JAK2 phosphorylation and downstream signaling as well as a reduction of *hTERT* and *STAT3* mRNA expression. Hence, our data demonstrate that imetelstat reduces TL and targets JAK/STAT signaling, particularly in CALR-mutated cells. Although the exact patient subpopulation who will benefit most from imetelstat needs to be defined, our data propose that CALR-mutated clones are highly vulnerable.

## Introduction

Myeloproliferative neoplasms (MPN) are a group of clonal hematopoietic disorders including polycythemia vera (PV), essential thrombocythemia (ET), and primary myelofibrosis (PMF), characterized by an excessive increase of granulomonocytic cells, erythroid cells, and/or platelets as well as different degrees of splenomegaly and bone marrow (BM) fibrosis (1). Driver mutations associated with MPN are found in genes of Janus kinase 2 (*JAK2*) (2–5), thrombopoietin receptor (*MPL*) (6, 7), or calreticulin (*CALR*) (8, 9). All mutations result in constitutive activation of JAK-STAT signaling, causing oncogenic transformation of these cells.

Telomeres are repetitive nucleoprotein structures at the end of most eukaryotic chromosomes and impact on the lifespan of a cell. They are maintained by the enzyme telomerase, which adds telomeric repeats (TTAGGG)n to the chromosome ends (10). The main components of telomerase are the reverse transcriptase hTERT (human telomerase reverse transcriptase) and an RNA component (human telomerase RNA, hTR). hTR contains a short sequence (5’-CUAACCCUAA-3’), which serves as a template in the reverse transcriptase reaction by hTERT and leads to telomeric repeat synthesis. The main observed functions of telomerase are to prevent the end-replication problem and to distinguish ends from double strand breaks. In adult somatic cells, telomerase is inactive, leading to telomere shortening, and subsequent cell-cycle arrest, apoptosis, or senescence. Most cancer cells have re-activated telomerase, which contributes to the infinite replication potential of these cells. These findings emphasize the rationale of blocking telomerase activity in cancer cells in order to reduce their replicative potential (11–13). This approach may be especially promising in BCR::ABL1-positive and -negative MPN, since CD34+ cells from these patients show both accelerated telomere shortening and upregulation of telomerase activity (14–17).

Imetelstat (GRN163L) is an oligonucleotide that blocks the enzymatic activity of hTERT by complementary binding to the template region of hTR (18, 19). The inhibition of telomerase activity and cell proliferation of oncogenic cell lines upon imetelstat treatment has been described for several non-hematological cancer cell lines and *in vivo* models (20–25) as well as for hematological malignancies such as acute myeloid leukemia (AML) (26), myelofibrosis (27) and myeloma (25). In MPN, a clinical phase 2 trial showed a high rate of hematologic responses in patients with ET upon imetelstat administration, accompanied by reduction of *JAK2*V617F variant allele frequency (VAF) after 3 months of administration (28). Moreover, 21% of patients with high-or intermediate-2-risk myelofibrosis (MF) showed complete or partial remission upon imetelstat treatment in a phase 1 trial (29). Recently, in the MYF2001 trial, imetelstat demonstrated clinical benefit in MF patients who had relapsed or were refractory to JAK inhibitors, with the data suggesting prolonged overall survival when compared to historical controls (30). The same study showed differences in response according to the genetic subtype, with a higher percentage of CALR mutant patients achieving a ≥ 25% VAF reduction than JAK2V617F mutant patients (30).

Further studies described that imetelstat is capable of selectively depleting MF hematopoietic stem and progenitor cells (HSPC) by inducing apoptosis *in vitro* (27). Additionally, it was shown that imetelstat hampers the formation of megakaryocytic colonies of ET patients’ mononuclear cells but not of healthy individuals, and reduces *hTERT* expression (31).

Here, we aimed to gain a deeper understanding of how telomerase inhibition by imetelstat induces responses in MPN. In this study, we describe a patient with advanced therapy-refractory PMF and used patient-specific induced pluripotent stem cells (iPSC) that recapitulated the complex mutational profile. By using these patient-derived cells, we analyzed the effects of imetelstat on cellular level as well as on hematopoietic cell development. In addition, to assess oncogene-specific effects of imetelstat, we tested murine and human cell lines expressing JAK2V617F or CALRdel52 and found driver oncogene-induced differences of imetelstat treatment on cell viability and oncogene-driven JAK-STAT signaling.

## Material and methods

### Patient data

After written informed consent, the patient was enrolled in the MYF2001 trial (NCT02426086), which was approved by the RWTH Aachen University Ethics Committee (EK 153/15). Peripheral blood samples were obtained from the MPN patient at the Department of Hematology, Oncology, Hemostaseology and Stem Cell Transplantation of RWTH Aachen University, or from fully anonymized healthy individuals at the Department of Transfusion Medicine at RWTH Aachen University, both after written informed consent, as approved by the local ethics committee (EK127/12, EK206/09 and EK099/14).

### Cell lines

Murine 32D cells (DSMZ, Braunschweig, Germany) were cultured in RPMI-1640 medium (PAN Biotec, Aidenbach, Germany) supplemented with 10% fetal calf serum (FCS) and 1% Penicillin-Streptomycin at 37°C with 5% CO_2_. Human TF-1 cells (DSMZ, Braunschweig, Germany) were cultured in RPMI-1640 medium supplemented with 20% FCS, 1% Penicillin and 2 ng/ml GM-CSF (Immunotools, Friesoythe, Germany). The transduction of 32D MPL-HA (32D^MPL^) cells with retrovirus containing JAK2V617F or CALRdel52 cDNA has been described before (Czech et al., 2019). The transduction of the TF1 cells was performed as follows: First, human TF1 cells were retrovirally transduced with the ecotropic Scl7a1 (Eco) receptor, which made them susceptible for infection with murine retroviruses, and cells were positively selected with neomycin. Retroviruses, which carried the pMSCV-MPL-HA vector, were produced using PlatE cells as previously described (32). Next, the TF1 Eco cells were transduced with a pMSCV-MPL-HA-generated (TF1^MPL^) retrovirus and positively selected with puromycin. Finally, the cells were transduced with retrovirus carrying either the JAK2V617F or CALRdel52 mutation.

### Drugs

Mismatch control (MM) and imetelstat were provided by Janssen, who was in co-development of imetelstat with Geron at that time, and dissolved in ultrapure water (vehicle).

### Protein lysates of cell lines

Cell lines were seeded at a density of 800,000 cells/ml or 1,500,000 cells/ml and stimulated as described in the figure legends. Afterwards, cells were harvested for protein lysates for Western blotting as described previously (32).

### iPSC generation and culture

Peripheral blood mononuclear cells (PBMCs) were obtained from the PMF patient enrolled in the MYF2001 trial and a healthy donor (HD) at the centralized Biomaterial Bank in Hospital RWTH Aachen or Transfusion Medicine in the University Hospital RWTH Aachen, respectively. Reprogramming of HD and PMF samples was performed as described previously (33, 34). After reprogramming, clonal iPSC lines were established by single cell seeding and manual individual colony picking. Stable iPSC lines were analyzed by next generation sequencing (NGS) for MPN-related mutations. iPSCs were cultured on matrigel-coated plates in StemMacs iPS Brew XF (Miltenyi Biotec, Bergisch Gladbach) and routinely passaged using Accutase (PAN Biotec, Aidenbach, Germany) or EDTA (Gibco, USA).

### Hematopoietic differentiation of iPSC

iPSC were differentiated into HSPC and megakaryocytes using an “spin-EB” differentiation protocol as described previously (35). Magnetic-activated cell sorting (MACS, Miltenyi Biotec, Bergisch Gladbach, Germany) using CD34 or CD61 beads were used to enrich for hematopoietic stem cells and megakaryocytes, respectively. To determine drug influence on megakaryocytic differentiation, 8 embryoid bodies (EB) were collected and cultured in SFM medium supplemented with compounds until day 14 of differentiation (35). The impact of imetelstat on megakaryopoiesis was evaluated by flow cytometry.

### Drug testing on HSCs and MKs

Isolated CD34+ and CD61+ cells on day 14 of differentiation were cultured for 72 h in SFM medium supplemented with drugs in 96-well format using 12,500 cells per well. Cell viability was assessed using a CellTiter-Glo Luminescent Cell Viability Assay (Promega, Madison, WI, USA).

### Colony formation (CFU) assay

iPSC-derived CD34+ cells or TF1^MPL^ cells were cultured in semi-solid medium supplemented with drugs in a density of 5,000 cells/ml or 1,000 cells/ml, respectively, for 12 days as described previously (35). After 12 days, colony numbers were counted, and cell types of the colonies were determined by flow cytometry analysis.

### Flow cytometry analysis

Cells were harvested and prepared for flow cytometry analysis as described previously (35). Stained cells were measured on a FACS Canto II (BD) and data were analyzed with FlowJo™ (version 10, Oregon, USA). Full list of antibodies is available in **Supplementary Table 1**.

### Measurement of telomere length (TL)

TL analysis was performed by flow-FISH as described previously (36, 37). Briefly, samples were stained with a telomere-specific (CCCTAA)3-peptide nucleic acid (PNA) FITC-labeled FISH probe (Panagene, Daejeon, Republic of Korea) for DNA hybridization combined with LDS 751 (Sigma Aldrich, St. Louis, Missouri, USA) for DNA counterstaining. Autofluorescence values of the respective unstained subpopulations were subtracted from the stained samples. The analysis was carried out using an FC-500 (Becton Dickinson, Franklin Lakes, New Jersey, USA) flow-cytometer. The mean TL was calculated relative to bovine thymocytes (internal control) with a known TL. The analysis was carried out in a single-blinded manner in triplicate.

### MTT assay

Metabolic activity as a measure of cell viability was analyzed using MTT as described previously (38). In short, both, 32D^MPL^ as well as TF1^MPL^ cells were applied into the MTT assay with 30,000 cells/100 µl medium per well. The metabolic process of tetrazolium reduction to formazan was measured 72 h after drug administration. This metabolic process (metabolic activity) reflects cell viability.

### SDS Page and western blotting

SDS Page and western blot analysis was performed as described before (32). Primary antibodies detecting pJAK2 (Y1007/1008) (#3771S), JAK2 (#32305S), pSTAT3 (Y705) (#9131S), STAT3 (#9139S) and pSTAT5 (Y695) (#9351L) were ordered from Cell Signaling (Leiden, Netherlands). Antibodies against STAT5 and GAPDH were purchased from Santa Cruz Biotechnology (Santa Cruz, CA, USA). Anti-rabbit and anti-mouse secondary antibodies conjugated to horseradish peroxidase were ordered from DAKO (Hamburg, Germany).

### RNA isolation and RT-qPCR

RNA was isolated according to manufacturer’s protocol (Qiagen, Hilden, Germany). For cDNA synthesis, 1 µg of RNA was used. Quantitative RT-qPCR was performed using the 7500 Fast Real time PCR System (Applied Biosystems by Life technologies, Paisly, UK) with the SYBR Selected Master Mix for CFX (Applied Biosystems). Target gene expression was calculated as percentage of *GAPDH* and illustrated as mean percentage of vehicle control (ddH_2_O). Primer Sequence: *STAT*3 f: CTCTGCCGGAGAAACAGGATGG, r: CTCTTGCAGGAAGCGGCTAT; *hTERT*: f: CGGAAGAGTGTCTGGAGCAA;r: GGATGAAGCGGAGTCTGGA; *GAPDH*: f: CCATCTTCCAGGAGCGAGATC, r: GCCTTCTCCATGGTGGTGAA.

### Statistical analysis

Graphical display and statistical analysis were performed with Prism 9 (GraphPad, San Diego, California, USA). Unless otherwise stated, all experiments were performed in triplicates and applied statistical tests are given in the figure legends. *p* values <0.05 were considered statistically significant (*p<0.05, **p<0. 01, ***p<0.001, ****p<0.0001).

## Results

### Beneficial clinical response to the telomerase inhibitor imetelstat in a patient with treatment-refractory PMF

**Figure 1:**
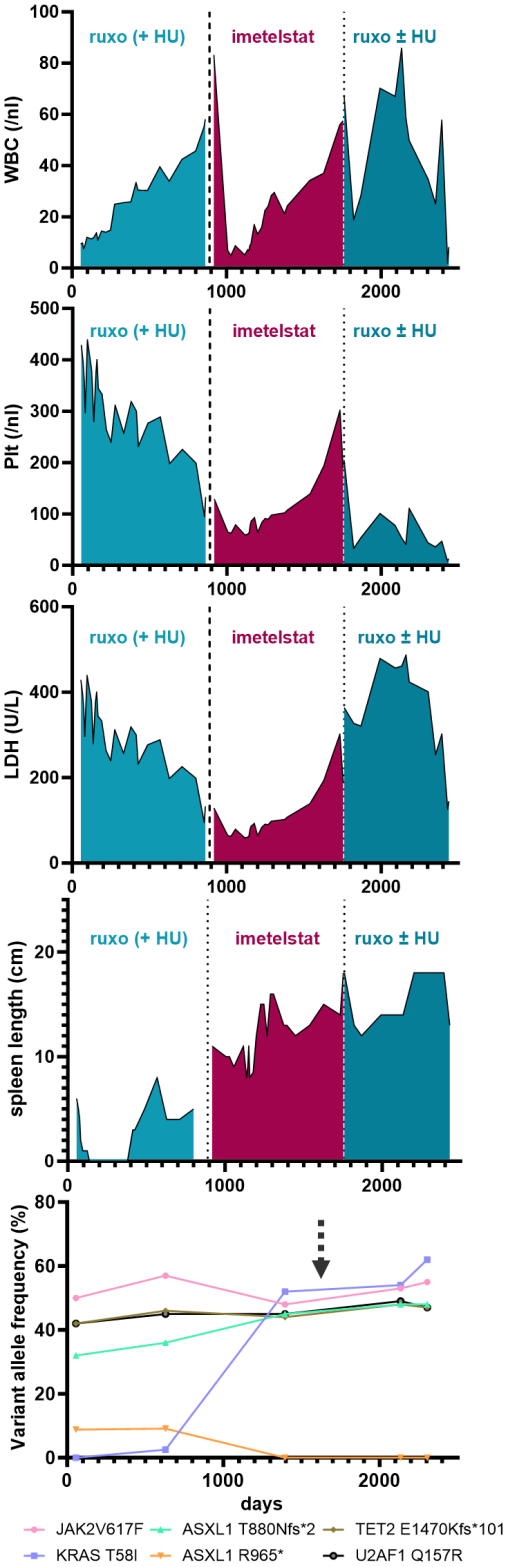
Blood parameters (i.e. WBC, Plt and LDH) and palpable spleen length (in cm below LCM - Left Costal Margin) are shown over time (days) during the course of the disease of the patient. Specific treatments are highlighted. The lowest graph illustrates the variant allele frequency of detected somatic mutations by next generation sequencing from peripheral-blood derived cells during the course of treatment. The arrow marks the time of induced pluripotent stem cell (iPSC) generation.

A 74 year-old female patient with newly-diagnosed JAK2V617F-positive PMF presented to our department after a 5-month treatment with hydroxyurea (HU) had not alleviated her pruritus and fatigue. At that time, her body weight was 74 kg, she had splenomegaly (palpable spleen 6 cm below the lower costal margin [LCM]), normal WBC and Hgb counts and slightly elevated platelet counts (429/nl) and LDH levels (283 U/L). Her blood differential showed 0-4% blasts, 3% basophils, 1% eosinophils, and 11% monocytes. BM histology was indicative of PMF, with an MF grade of 2 (scale 0-3). Initially, NGS of the peripheral blood showed the presence of two ASXL1 mutations, a TET2 mutation, and a U2AF1 mutation, and a low-frequency KRAS mutation (**Figure 1**, **Supplementary Table 2**). After several lines of treatments, including ruxolitinib treatment, the patient´s disease progressed, and two years after her initial presentation, when her WBC rose up to 50/nl (while Hgb and platelet counts were normal) and she developed increased sweating, HU was added (500 mg QD PO) to ruxolitinib treatment. Four months later, which included dose adjustments of both ruxolitinib (15 mg BID) and HU (500 mg BID), the PMF had progressed, with increasing splenomegaly (11 cm below LCM), leukocytosis (56.9/nl), anemia (10.7 g/dl), and thrombocytopenia (145/nl), elevated LDH (656 U/L), as well as pruritus, sweating, and reduced appetite. A dose increase of ruxolitinib was not tolerated.

**Figure 2:**
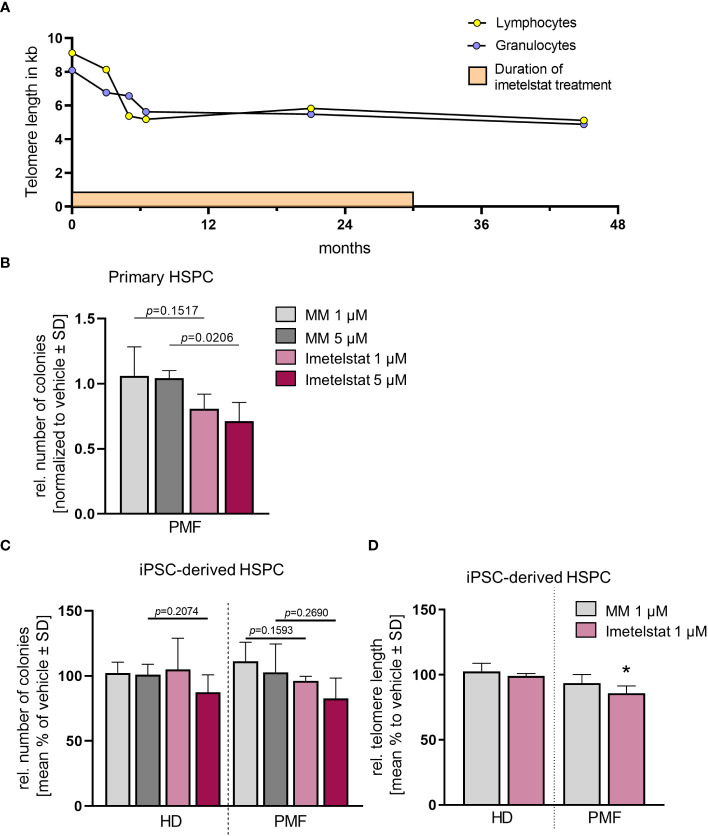
**(A)** Telomere length (TL) of a PMF patient treated with imetelstat for 30 months in the MYF2001 clinical trial was measured via flow-FISH in the granulocytic and the lymphocytic cell population. **(B)** Relative colony number in a CFU assay of primary CD34+ cells treated with 1 µM or 5 µM of MM or imetelstat. Colony number was normalized to vehicle control. *n*=3. For statistical analyses, t-test was performed to compare 1 µM or 5 µM MM to 1µM or 5 µM imetelstat, respectively. *P* values are given. **(C)** Relative colony number of iPSC-derived CD34+ cells of HD control or PMF in a CFU assay treated with 1 µM or 5 µM MM or imetelstat. Relative colony numbers were normalized to vehicle control for each condition. For statistical analyses, t-test was performed. *n*=3. **(D)** Telomere length of iPSC-derived CD34+ HD or PMF cells treated with 1 µM MM, 1 µM imetelstat or vehicle for 14 days. Telomere length is shown relative to vehicle control. Flow cytometry analysis was used to calculate telomere length. To calculate significance, t-test was performed. *p<0.05, *n*=3. HD, healthy donor; MM, mismatch control; PMF, primary myelofibrosis; SD, standard deviation. HD, healthy donor; HSPC, hematopoietic stem and progenitor cells; iPSC, induced pluripotent stem cell; MM, mismatch control; SD, standard deviation.

Thus, failure to combined treatment with ruxolitinib and HU was diagnosed, and the patient was enrolled in the MYF2001 phase II multicenter clinical trial, conducted in patients with intermediate-2 or high-risk myelofibrosis JAKi R/R (JAK inhibitor relapsed/refractory) (ClinicalTrials.gov identifier: NCT02426086). Intravenous treatment with 9.4 mg/kg imetelstat was initiated, and within three months, WBC counts, hemoglobin and LDH levels normalized. Telomere length (TL) decreased rapidly during the first six months of imetelstat treatment and remained low (8.09 kb to 5.49 kb in granulocytes after 21 months; **Figure 2A**). There were no more blasts in the PB, and left shift had essentially disappeared. Platelet counts decreased to around 70/nl and remained stable afterwards, and no hemorrhagic complications occurred. The weight of the patient had decreased to 62 kg, mostly due to nausea and inappetence. Thrombocytopenia, nausea, and weight loss were attributed to imetelstat treatment. The spleen size had decreased to 8 cm below LCM, and BM histology showed stable PMF, but no signs of acute leukemic transformation. Corticosteroids were successfully administered to treat the nausea.

During the subsequent months, there was evidence of clonal evolution (NGS now showed disappearance of the *ASXL1* R965* mutation but an increase of the KRAS mutant allele burden to 52%) (**Figure 1**, **Supplementary Table 2**, year 5), but the patient remained clinically stable and on study for a total duration of 2.5 years. After that time, she had to be taken off study due to progressive thrombocytopenia and leukocytosis, splenomegaly (increase to 18 cm below LCM and new-onset generalized pain), cachexia (50 kg), and increasing night sweats. At that time, NGS analysis was essentially the same as two years before (**Supplementary Table 2**, year 7). She was re-started on HU and ruxolitinib treatment, but she became increasingly frail and died two years later (year 9), shortly after the diagnosis of post-MF AML. In summary, while imetelstat did not cure PMF, it led to clinical improvement for over 2.5 years and extension of expected overall survival in this patient with very high-risk therapy-refractory PMF.

### Imetelstat specifically targets telomere length in malignant iPSC-derived cells

To elucidate disease-modifying activity of imetelstat in MPN, we used PBMC of this PMF patient or a HD as a non-disease control to generate iPSC. Genetic analysis of the generated PMF iPSC showed the same allele burden for MPN-related mutations as the peripheral blood of the PMF patient, including JAK2V617F and KRAST58I mutations (**Supplementary Table 2**).

The patient- as well as the HD-derived iPSC were differentiated toward CD34+ HSPC and CD61+ MK, as described previously (35). First, we investigated whether imetelstat impaired the growth and colony forming unit (CFU) potential of CD34+ cells derived directly from the PMF patient or from iPSC in clonogenic assays (**Figures 2B, C**). Primary PMF patient cells showed a decrease in the number of colonies when treated with 5 µM imetelstat compared to 5 µM MM control (**Figure 2B**). In contrast, in iPSC-derived control cells (HD) and PMF cells, neither exposure to 1 or 5 µM MM nor imetelstat reduced the number of colonies (**Figure 2C**), demonstrating that imetelstat hampers cell proliferation and colony growth of bulk cells but not of the clonal iPSC-derived CD34+ cells.

Although colony growth of iPSC-derived HSPC was not altered by imetelstat, this treatment may have an effect on hematopoietic differentiation and cell composition. Hence, we analyzed whether imetelstat affected the development of different hematopoietic progenitors, such as granulocytic and monocytic progenitor cells, in CFU assays. Cells were collected from semi-solid medium and analyzed by flow cytometry to identify hematopoietic subpopulations (**Supplementary Figures 1A, B**). Overall, imetelstat treatment had no impact on HD- or PMF-derived granulocytes, monocytes, or macrophages **Supplementary Figure 1C**).

The efficacy of imetelstat in malignant megakaryocytes has been described previously (27, 31, 39). Therefore, the impact of imetelstat on megakaryocytic development was investigated. The percentage of HD-iPSC- vs. PMF-iPSC-derived CD41+/CD61+ megakaryocytes was analyzed on day 14 of differentiation, after 6 days of treatment with 1 and 5 µM MM or imetelstat or vehicle control (**Supplementary Figures 1A**, **2A**). When compared to control, the percentage of megakaryocytes was not changed, and no loss in cell viability was observed in either HD- or PMF-derived megakaryocytes or CD34+ cells after 3 days of treatment (**Supplementary Figures 2B, C**).

To investigate the activity of imetelstat in the iPSC-derived CD34+ HSPC, we determined whether imetelstat induced a reduction of TL in these cells after two weeks of *in vitro* treatment. TL, which was overall increased during the reprogramming process (from 5.8 kb and 5.49 kb in lymphocytes and granulocytes, respectively, to 13.9 kb ± 1.85 in iPSC-derived HSPC of the patient), was assessed by flow-cytometric analysis in HD and PMF cells. HD cells showed a similar TL, regardless of whether they were treated with vehicle or 1 µM MM or imetelstat (**Figure 2D**). Importantly, imetelstat treatment caused a reduction of TL in PMF cells compared to MM control (loss of 14.02% ± 5.42; *p*=0.011). Noteworthy, TL reduction of 10% is considered to reflect a reduction of approximately 20 years of cellular aging (40). Our data demonstrate that imetelstat selectively reduced TL of the malignant but not non-malignant HD cells derived from iPSC, which is in line with the effect on TL observed in the patient (**Figure 2A**).

Thus, together, our data demonstrate that while imetelstat directly reduced TL of mutated iPSC-derived CD34+ cells, short term *in vitro* treatment of imetelstat did not lead to reduced clonogenic capacity of these cells, suggesting that TL was not critically short in these cells, potentially because of TL increase during iPSC reprogramming. However, treatment of primary CD34+ cells from the patient with imetelstat significantly reduced their clonogenic growth, implying that TL was critically short in these cells and confirming the potential of imetelstat to inhibit these cells *in vivo*.

### Rapid targeting of human TF1^MPL^ JAK2V617F cells by imetelstat

While the patient´s response to imetelstat was encouraging, this was not reflected in the response of iPSC-derived HSPCs. Hence, we aimed to analyze JAK-STAT signaling upon short imetelstat treatment in cells which differ solely in JAK2V617F mutation (by using JAK2V617F- vs. empty vector-transduced TF-1^MPL^ cells).

**Figure 3:**
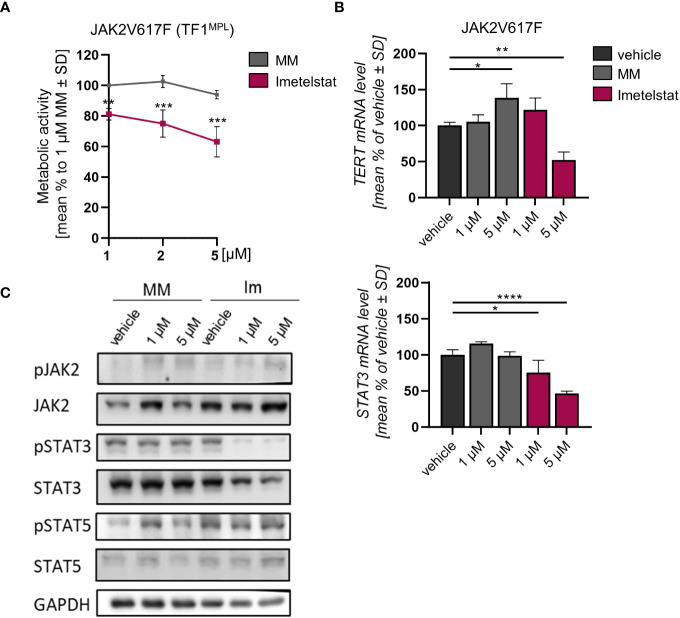
**(A)** MTT assay of TF1^MPL^ cells expressing JAK2V617F exposed to 1, 2 and 5 µM MM or imetelstat for 72 (h) n=3. Each cell mean was compared to the other cell mean of the row in a Two-Way ANOVA multiple comparison test (Bonferroni). **(B)** *TERT* and *STAT3* mRNA expression in JAK2V617F expressing TF-1^MPL^ cells upon MM control or imetelstat stimulation for 6 (h) Expression was calculated as percentage of vehicle control normalized to *GAPDH*. n=3. T-test was performed. *p<0.05, **p<0.01, ***p<0.001, ****p<0.0001. MM, mismatch control; SD, standard deviation. **(C)** Western blotting and indicated immunostainings of TF-1^MPL^ cells expressing JAK2V617F after vehicle, MM control or imetelstat treatment for 24h. GAPDH was used as loading control. *n*=2. Im; imtetelstat.

The effect of imetelstat on cell viability of human TF-1^MPL^ cells, stably expressing JAK2V617F was assessed by MTT assays. These TF-1^MPL^ cells have acquired growth factor-independence due to the presence of the JAK2V617F oncogene. Cell viability was significantly reduced in a concentration-dependent manner in JAK2V617F cells (down to 63.1% ± 9.99) compared to 1 µM MM control (**Figure 3A**).

In diverse tumors, high *hTERT* expression correlates with increased telomerase activity (41, 42). Hence, we next analyzed whether *hTERT* expression is affected by imetelstat treatment. In JAK2V617F-mutated cells, while *STAT3* expression was decreased upon 1 µM and 5 µM imetelstat exposure, *hTERT* expression was reduced only by 5 µM imetelstat (**Figure 3B**). Unexpectedly, *hTERT* expression was significantly upregulated by 5 µM MM control. To elucidate whether the reduction of cell viability upon imetelstat treatment was mediated via the oncogene-driven JAK-STAT pathway, we evaluated alterations in JAK2V617F downstream signaling by Western blotting upon 24 h of imetelstat or MM control treatment. Interestingly, while JAK2 phosphorylation was unchanged, we observed a reduction of pSTAT3 as well as overall STAT3 protein (**Figure 3C**) upon 1 and 5 µM imetelstat treatment, which was in line with the reduced expression of *STAT3* mRNA. Meanwhile, neither STAT5 phosphorylation nor STAT5 total protein were altered.

### Imetelstat shows stronger effects on human *CALR*del52- than *JAK2*V617F-mutated cells and acts via the JAK-STAT axis

Although clinical data of imetelstat-treated patients carrying CALR frameshift mutations exist and suggest clinical efficacy (see above), little is known about mechanistical differences of JAK2- vs CALR-mutated cells. Therefore, we studied whether imetelstat targets CALRdel52-mutant cells, the second most frequent mutation in MPN, as efficiently as JAK2V617F-mutated cells.

**Figure 4:**
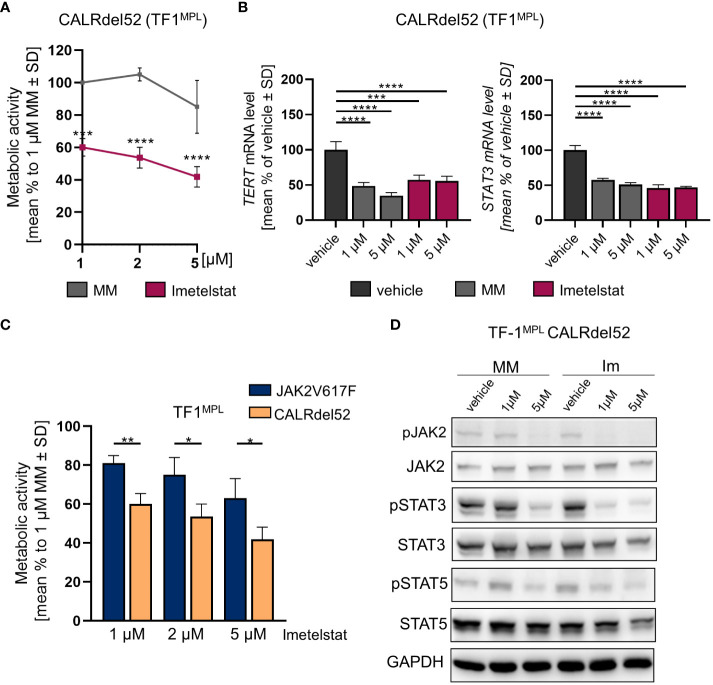
**(A)** MTT assay of TF1^MPL^ cells expressing CALRdel52 exposed to 1, 2 and 5 µM MM or imetelstat for 72 (h) n=3. Each cell mean was compared to the other cell mean of the row in a Two-Way ANOVA multiple comparison test (Bonferroni). **(B)** *TERT* and *STAT3* mRNA expression in CALRdel52 expressing TF-1^MPL^ cells upon MM control or imetelstat stimulation for 6 (h) Expression was calculated as percentage of vehicle control normalized to *GAPDH*. n=3. T-test was performed. *p<0.05, **p<0.01, ***p<0.001, ****p<0.0001. MM: mismatch control; SD: standard deviation. **(C)** Metabolic activity of TF1^MPL^ cells expressing CALRdel52 or JAK2V617 stimulated with different concentrations of imetelstat for 72 (h) n=3. T-test was performed. **(D)** Western blotting and indicated immunostainings of TF-1^MPL^ cells expressing CALRdel52 after vehicle, MM control or imetelstat treatment for 24h. GAPDH was used as loading control. *n*=3. Im; imtetelstat.

First, growth factor independent TF-1^MPL^ CALRdel52 cells were generated, and MTT assays were performed upon *in vitro* treatment with 1, 2, or 5 µM of imetelstat or MM control. Cell viability was strongly and significantly reduced already with 1 µM of imetelstat (**Figure 4A**). Second, gene expression of *hTERT* and *STAT3* was analyzed, with CALRdel52 cells showing a strong decrease in *hTERT* expression when treated for 6 h with imetelstat or MM (1 and 5 µM, respectively) compared to vehicle alone (**Figure 4B**), and the same pattern was observed for *STAT3* expression in these cells.

Next, we compared the two cell lines, JAK2V617F vs. CALRdel52 TF-1^MPL^ cells, upon imetelstat treatment. Interestingly, imetelstat-induced reduction of cell viability after 72 h was significantly more pronounced in CALRdel52 cells as compared to JAK2V617F cells, when treated with 1 µM, 2 µM or 5 µM imetelstat, suggesting that cell survival of malignant CALRdel52 cells may be more dependent on telomerase activity or off-target effects compared to JAK2V617F cells (**Figure 4C**).

These results were additionally confirmed in murine 32D^MPL^ cells harboring the JAK2V617F or CALRdel52 mutation (43, 44) (**Supplementary Figures 3A, B**).

Remarkably, Western blot analysis in TF-1^MPL^ CALRdel52 cells validated a decrease in the phosphorylation of JAK2, STAT3, and STAT5 following imetelstat treatment (**Figure 4D**). This observation aligns with the findings from the analysis of *STAT3* and *hTERT* expression (**Figure 4B**), as well as the alterations in metabolic activity observed in TF-1^MPL^ (**Figure 4A**) and 32D^MPL^ CALRdel52 cells (**Supplementary Figure 3A**). Notably, even though MM control at 5 µM exhibited a reduction in pJAK2, pSTAT5, and pSTAT3, the impact of imetelstat on the phosphorylation of JAK-STAT proteins was more pronounced.

As we observed a swift effect of imetelstat on dephosphorylation of JAK2 in CALRdel52-mutant TF-1^MPL^ cells, we also assessed JAK-STAT signaling in the 32D clones and confirmed the fact that JAK2 and pJAK2 protein levels were decreased by MM or imetelstat treatment in CALRdel52 but not JAK2V617F cells (**Supplementary Figure 3C**). Overall, the data demonstrate that imetelstat treatment reduces JAK2 signaling in CALRdel52- but less pronounced in JAK2V617F-mutant 32D^MPL^ cells.

To confirm that overexpression of JAK2 (wildtype or mutant JAK2V617F) is not responsible for the differential response of CALRdel52- vs. JAK2V617F cells to imetelstat, 32D^MPL^ CALRdel52 cells ectopically expressing JAK2 wildtype (WT) were assessed by MTT assay. We confirmed significant reduction of cell viability in 32D^MPL^ JAK2 WT CALRdel52 cells upon treatment with 5 µM imetelstat but not the MM control (**Supplementary Figure 4A**). This shows that overexpression of wildtype JAK2 does not counteract the sensitivity of CALRdel52 32D^MPL^ cells to imetelstat and points to a specific vulnerability of CALR mutant cells. In addition, JAK2 expression partially prevented 5 µM MM-induced loss of viability.

As a reduction of pJAK2 in 32D^MPL^ CALRdel52 cells was observed, the phosphorylation pattern of the JAK2 downstream targets, STAT3 and STAT5, was analyzed in the 32D^MPL^ JAK2 WT CALRdel52 cells upon imetelstat treatment by Western blotting (**Supplementary Figure 4B**). Densitometry analysis confirmed the reduction of JAK2 phosphorylation when cells were treated with 1 µM or 5 µM of imetelstat as well as MM in a dose-dependent manner compared to vehicle control (**Supplementary Figure 4C**). In addition, phosphorylation of STAT3 and STAT5 were also decreased when cells were exposed to 1 µM or 5 µM imetelstat but not the MM control.

In conclusion, these data demonstrate that imetelstat treatment leads to reduction of downstream JAK signaling in CALRdel52, but not JAK2V617F cells, while TL shortening was explicitly demonstrated in the JAK2V617F PMF patient and iPSC-derived HSPC of this patient (**Figure 2**).

## Discussion

In the present study, we demonstrate clinical activity of the telomerase inhibitor imetelstat in a patient with advanced PMF. The emergence of a RAS-mutant clone during ruxolitinib treatment may have been relevant for the final loss of activity of imetelstat. Furthermore, ASXL1 and U2AF1 (particularly Q157R) mutations have been included among the high-molecular risk mutations in MF due to their association with shorter overall survival (45). Our data show that the RAS mutant clone emerged during ruxolitinib treatment (2.53%) when splenomegaly was still rather mild and then expanded over the course of the next years both during imetelstat (52%) and ensuing ruxolitinib +/-HU treatment (62%). RAS mutations are indeed predictors of a reduced response to JAK inhibitors, and patients carrying a RAS mutation have an increased risk of leukemic transformation (46–48). Moreover, a recent study by Maslah and colleagues describes a clonal selection of RAS mutated clones mediated by JAK inhibition (49). Hence, the RAS clone was most likely already selected during ruxolitinib treatment and later expanded despite imetelstat treatment. Notably, we currently lack sufficient clinical data regarding imetelstat treatment in MPN or PMF to confirm selection of RAS mutated clones. However, in a recent manuscript (made available on bioRxiv in April 2023) by Stephen Lane’s group, the authors reported a preference for imetelstat to target NRAS mutated clones in a pre-clinical AML PDX mouse model (50).

In addition, we used iPSC of this patient to evaluate potential imetelstat-associated disease-modifying effects. Imetelstat showed significant activity on PMF patient-derived CD34+ HSPC, but neither on iPSC-derived HSPC from healthy controls nor the PMF patient. We did not observe any impact of imetelstat on megakaryopoietic differentiation or viability *in vitro*, which contrasts with the study of Mosoyan et al, who reported that imetelstat selectively inhibits megakaryopoesis of MPN samples by preventing megakaryocyte maturation and reducing secretion of fibrinogenic factors (39). Mosoyan and colleagues also reported that imetelstat delays megakaryocyte precursors maturation in non-malignant hematopoiesis (39). In this study, iPSC-derived HSPC were used and, no statistically significant effects of imetelstat were observed on colony formation. However, efficacy of imetelstat on CD34+ cells isolated directly from the PMF patient was shown, which is consistent with the results by Mosoyan et al.

As we observed effects of MM control especially in CALR mutated cells, we have used lower imetelstat concentrations, ranging from 1 to 5 µM, than other *in vitro* studies, which used concentrations of up to 15 µM, and effects of imetelstat on cell viability and megakaryocytic differentiation were reported for concentrations of 7.5 µM or higher (27, 31, 39). Our data clearly demonstrate that an imetelstat concentration of 1 µM is sufficient to selectively target telomerase activity and induce telomere shortening in MPN mutant cells, as shown by a reduction of TL by 10% in PMF iPSC-derived CD34+ compared to HD cells. Meanwhile, we observed an overall increase of TL in the iPSC-derived CD34+ cells due to reprogramming in comparison to the primary patient cells extracted during the clinical treatment course. This TL increase may explain the low effect of imetelstat on iPSC-derived hematopoietic cells, as longer TL may reduce response to imetelstat and vice versa (51, 52).

Furthermore, in order to address potential molecular mechanisms of imetelstat bioactivity, we investigated whether the response to imetelstat was dependent on the type of MPN driver mutation (JAK2V617F vs. CALRdel52). Tefferi et al. had described that the clinical response to imetelstat was exclusively seen in patients with a JAK2V617F mutation, with 27% of JAK2V617F-positive vs. 0% of JAK2V617F-negative patients responding (29). Our *in vitro* experiments clearly demonstrate that metabolic activity was reduced in both JAK2V617F and CALRdel52 TF1^MPL^ cells. Our data are supported by other groups showing that imetelstat acted irrespective of the driver mutation and is able to reduce the allele burden of patients with *JAK2*, *CALR*, or *MPL* mutations (27, 30).

Nevertheless, our results in TF-1 and 32D cells show that the effects were even more pronounced in CALRdel52 vs. JAK2V617F cells, which matches more recent clinical data from the MYF2001 trial, showing that the percentage of patients who experienced a ≥ 25% allele burden reduction with imetelstat was higher in the CALR- vs. JAK2-mutant group of patients (100% vs. 31% on 9.4 mg/kg imetelstat) (30). In further experiments, assessing potential mechanisms for this difference, we observed a reduction in phosphorylated JAK2 protein in CALRdel52 cells in contrast to JAK2V617F cells at 1 µM, and, even more pronounced, at 5 µM imetelstat. A relationship of JAK-STAT activation and telomerase regulation has already been described, with STAT3 regulating *hTERT* expression (53). These data are in line with our findings showing the same pattern of *hTERT* and *STAT3* reduction upon imetelstat-mediated JAK-STAT signaling inactivation in CALRdel52 cells. While our *in vitro* results suggest inhibition of pJAK2 by imetelstat treatment, the underlying mechanisms for this effect still need to be elucidated. Telomere-independent on-target non-canonic or off-target effects may play a role, as already suggested by Hidaka and colleagues (54). Imetelstat is a lipid-conjugated oligonucleotide. Possible off-target effects relying on the oligonucleotide structure of imetelstat were already studied because of oligonucleotide-mediated activation of Toll-like receptors (TLR) as part of the innate immune system (55). Although imetelstat mediated activation of TLR was ruled out in this study, it cannot be excluded that there are other imetelstat driven on- or off-target effects, especially since, even in the clinical trials, the effects of imetelstat on platelet counts were rather rapidly occurring effects (29). Similarly, we observed a rapid reduction of TL in *in vitro* treatment of iPSC-derived HSPC. This effect may be explained by end resection and activity of exonuclease 1, a mechanism involved in regular repair of DNA double-strand breaks and telomere elongation (56), but, here, imetelstat-driven effects need to be considered.

We show a reduction of cell viability of TF-1^MPL^ and 32D^MPL^ cell lines upon imetelstat treatment, suggesting that the reported effect on cell viability is not mediated trough long-term inhibition of the telomerase activity, but rather through a more immediate process. Of note, telomerase inhibition itself is not sufficient to induce cell death of tumor cells, but short telomeres trigger DNA-damage response that induce cell death (57). In our experiments, we demonstrate that imetelstat but not the MM control reduced TL in malignant hematopoietic cells after 14 days *in vitro* treatment, but sufficient telomere shortening to induce cell death after 3 days of treatment is doubtful. Interestingly, MM (sequence mis-matched oligonucleotide) showed in part similar efficacy as imetelstat in reducing *STAT3* and *hTERT* expression (TF-1 cells), as well as reduction of viability (32D cells) in CALRdel52 but not in JAK2V617F cells. The MM effect was abolished by overexpression of JAK2 WT in CALRdel52-positive 32D cells. Hence, oligonucleotide-mediated off-target effects may be more pronounced in CALR-mutated cells. There are only scarce data describing the ability of wildtype CALR to bind to oligonucleotides/mRNA (58). But it can be hypothesized that mutated CALR gains novel binding properties, as prominently exemplified by TPOR binding and, as a result, TPOR activation in MPN (59, 60). While our findings regarding the oncogene-specific efficacy of imetelstat and the swift downregulation of pJAK2 in CALRdel52 expressing cells upon imetelstat treatment align well with the MYF2001 clinical trial data, our study lacks confirmation of the direct inhibitory effect on signaling events in primary patient samples (specifically comparing JAK2V617F to CALRdel52), and these results thus require clinical verification in subsequent studies.

Collectively, we demonstrate here that imetelstat reduces TL selectively in malignant but not healthy cells, which may eventually lead to telomere–mediated replicative exhaustion of affected neoplastic (stem) cells. However, whether imetelstat exerts its effect via inhibition of the canonical or non-canonical telomerase pathway, or via yet undefined off-target effects such as overall changes in genome stability, is currently unresolved, particularly with respect to the different MPN subtypes. Even so, our data clearly implicate the oncogene-driven JAK-STAT pathway as an additional target of imetelstat.

## Data availability statement

The original contributions presented in the study are included in the article/**Supplementary Material**. Further inquiries can be directed to the corresponding authors.

## Ethics statement

The studies involving humans were approved by RWTH Aachen University Ethics Committee - Pauwelsstr.30 -52074 Aachen - Germany. The studies were conducted in accordance with the local legislation and institutional requirements. The participants provided their written informed consent to participate in this study.

## Author contributions

KO: Conceptualization, Methodology, Visualization, Writing – review & editing, Formal Analysis, Investigation, Writing – original draft. BA: Conceptualization, Formal Analysis, Investigation, Methodology, Visualization, Writing – review & editing. Md: Formal Analysis, Investigation, Methodology, Writing – review & editing. AM: Formal Analysis, Investigation, Writing – review & editing, Data curation. AA: Formal Analysis, Investigation, Writing – review & editing, Methodology. FB: Methodology, Writing – review & editing, Resources. DG: Resources, Writing – review & editing. SI: Resources, Writing – review & editing. KP: Resources, Writing – review & editing. TB: Resources, Writing – review & editing. MZ: Resources, Writing – review & editing. NC: Writing – review & editing, Conceptualization, Funding acquisition, Methodology, Supervision, Visualization. SK: Conceptualization, Methodology, Resources, Supervision, Writing – review & editing.
