# Supplementary material for: The telomerase inhibitor imetelstat differentially targets JAK2V617F versus CALR mutant myeloproliferative neoplasm cells and inhibits JAK-STAT signaling
Source: Front Oncol. 2023 Oct 24;13:1277453. doi: 10.3389/fonc.2023.1277453 (PMC10628476; doi:10.3389/fonc.2023.1277453)
Supplement: Supplementary file 1 [file DataSheet_1.docx]

**The telomerase inhibitor imetelstat differentially targets JAK2V617F versus CALR mutant myeloproliferative neoplasm cells and inhibits JAK-STAT signaling**

Kathrin Olschok^1,2^, Bianca Altenburg^1,2^, Marcelo A. S. de Toledo^1,2^, Angela Maurer^1,2^, Anne Abels^1,2^, Fabian Beier^1,2^, Deniz Gezer^1,2^, Susanne Isfort^1,2^, Katrin Paeschke^2,3^, Tim H. Brümmendorf^1,2^, Martin Zenke^1,2^, Nicolas Chatain^1,2, †^, and Steffen Koschmieder^1,2, †^

^1^Department of Hematology, Oncology, Hemostaseology, and Stem Cell Transplantation, Faculty of Medicine, RWTH Aachen University, Pauwelsstraße 30, 52074 Aachen, Germany

^2^Center for Integrated Oncology Aachen Bonn Cologne Düsseldorf (CIO ABCD), Germany

^3^Institute of Clinical Chemistry and Clinical Pharmacology, University Hospital Bonn, 53127 Bonn, Germany

^†^These authors share senior authorship

Correspondence: Prof. Dr. med. Steffen Koschmieder; E-mail: skoschmieder@ukaachen.de; and Dr. rer. nat. Nicolas Chatain; E-mail: nchatain@ukaachen.de

**Short title:** Imetelstat preferentially targets CALR mutant cells and signaling

*Supplementary data*


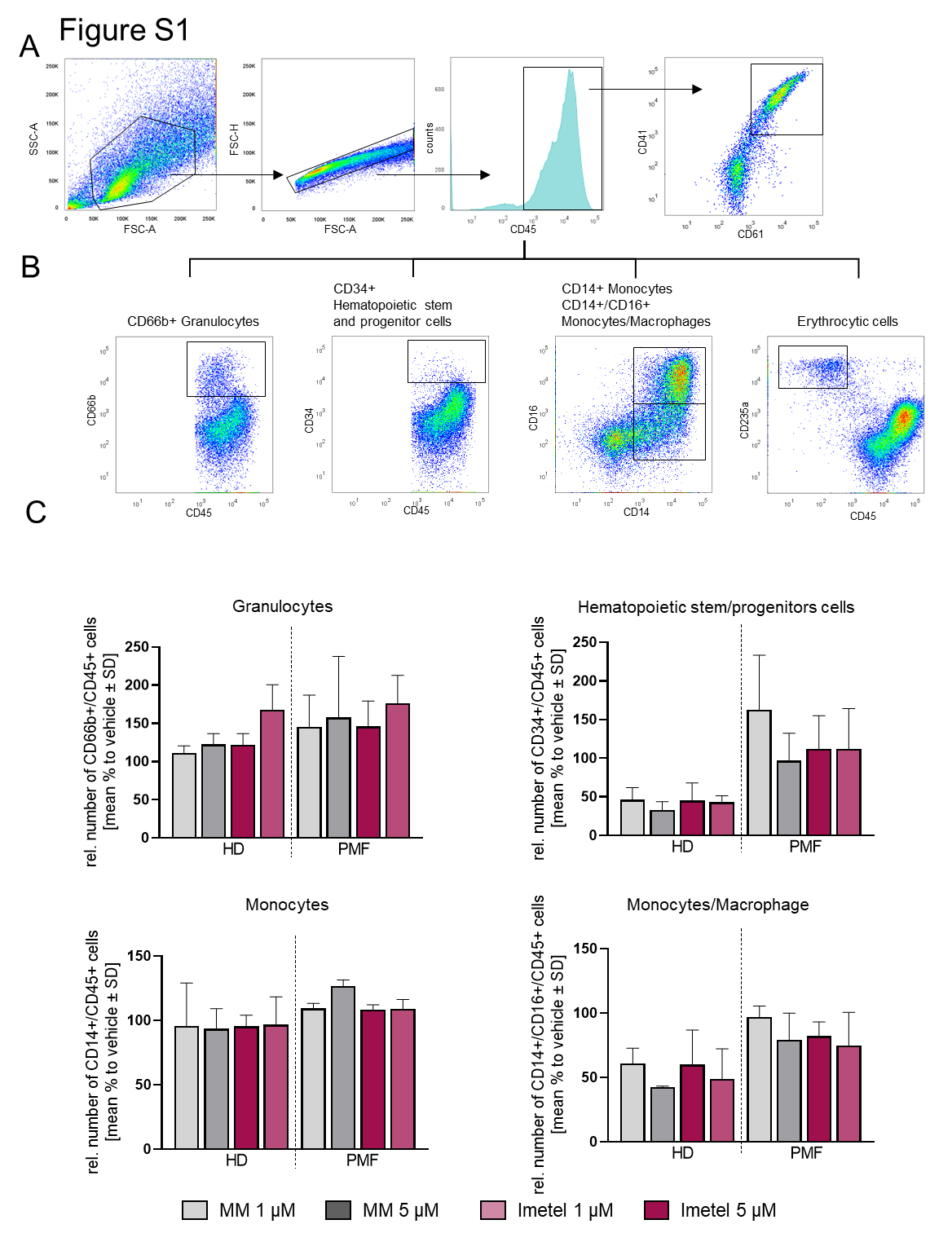


**Figure S1.** (A) Representative gating strategy to evaluate number of CD41/CD61+ megakaryocytic cells after 14 days of differentiation. Living cells were first gated on forwards scatter (FSC-A)/side scatter (SSC-A) and further gated on single cells and CD45+ cells before setting the gates on CD41+/ CD61+ megakaryocytes. (B) Representative gating strategy to identify hematopoietic subpopulations in CFU assays by flow cytometry analysis. Gates for typical surface markers was set on the living, CD45 expressing single cell population. (C) Relative number of hematopoietic subpopulations in CFU assays treated with 1 µM or 5 µM MM or imetelstat compared to 1 µM MM. iPSC‑derived CD34+ cells of HD control or PMF were cultured in semisolid medium for 12 days. Hematopoietic progenitors, granulocytes, monocytes, macrophages, and erythrocytic cells were determined by flow cytometry analysis. **p*<0.05, *n*=3. HD: healthy donor; Imetel: Imetestat; MM: mismatch control; SD: standard deviation


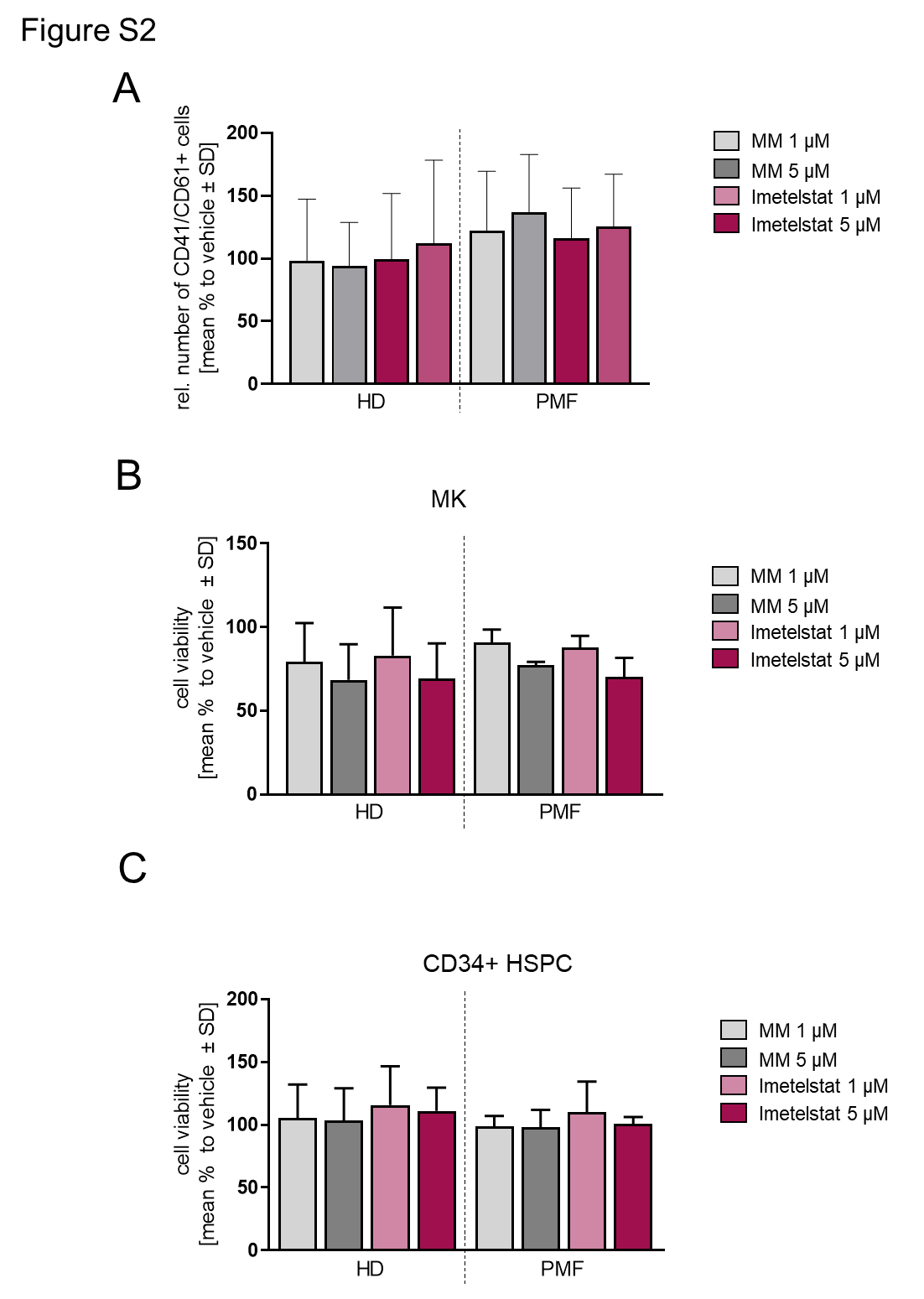


**Figure S2.** (A) Number of CD41+/CD61+ megakaryocytic cells in HD control or MPN iPSC after 6 days of treatment with vehicle control, 1 or 5 µm MM or imetelstat during differentiation. Number of megakaryocytic cells was compared to vehicle control. *n*=3. (B) Drug response of control HD or PMF iPSC‑derived CD61+ megakaryocytes treated with 1 or 5 µM MM or imetelstat for 72 h. Vehicle‑treated cells were used as control **p*<0.05, ***p*<0.01, *n*=3. (C) Drug response of control HD or PMF iPSC‑derived CD34+ HSPC treated with 1 or 5 µM MM or imetelstat for 72 h. Vehicle‑treated cells were used as control. *n*=3. HD: healthy donor; HSPC: hematopoietic stem and progenitor cells; iPSC: induced pluripotent stem cell; MK: megakaryocytes; MM: mismatch control; SD: standard deviation


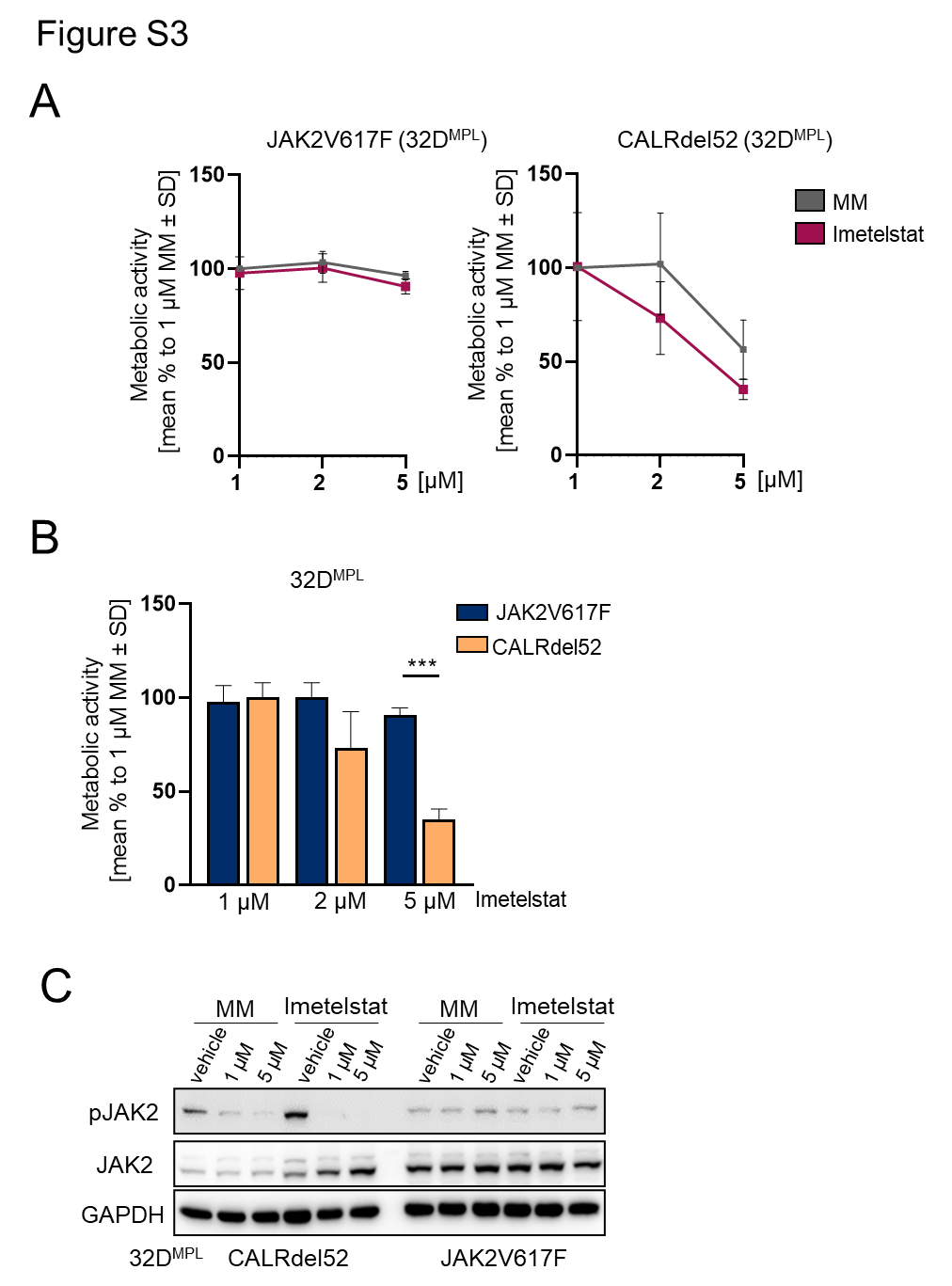


**Figure S3.** (A) MTT assay of 32D^MPL^ cells expressing CALRdel52 or JAK2V617F mutation exposed to 1, 2 and 5 µM MM or imetelstat for 72 h. *n*=3. (B) Metabolic activity of 32D^MPL^ cells expressing CALRdel52 or JAK2V617 treated with different concentrations of imetelstat for 72 h. n=3. T-test was performed. ****p*<0.001. (C) Western blot analysis of indicated 32D^MPL^ cells treated with vehicle, 1 µM or 5 µM MM control, or imetelstat for 24 h stained for JAK2 and pJAK2. GAPDH served as a loading control.


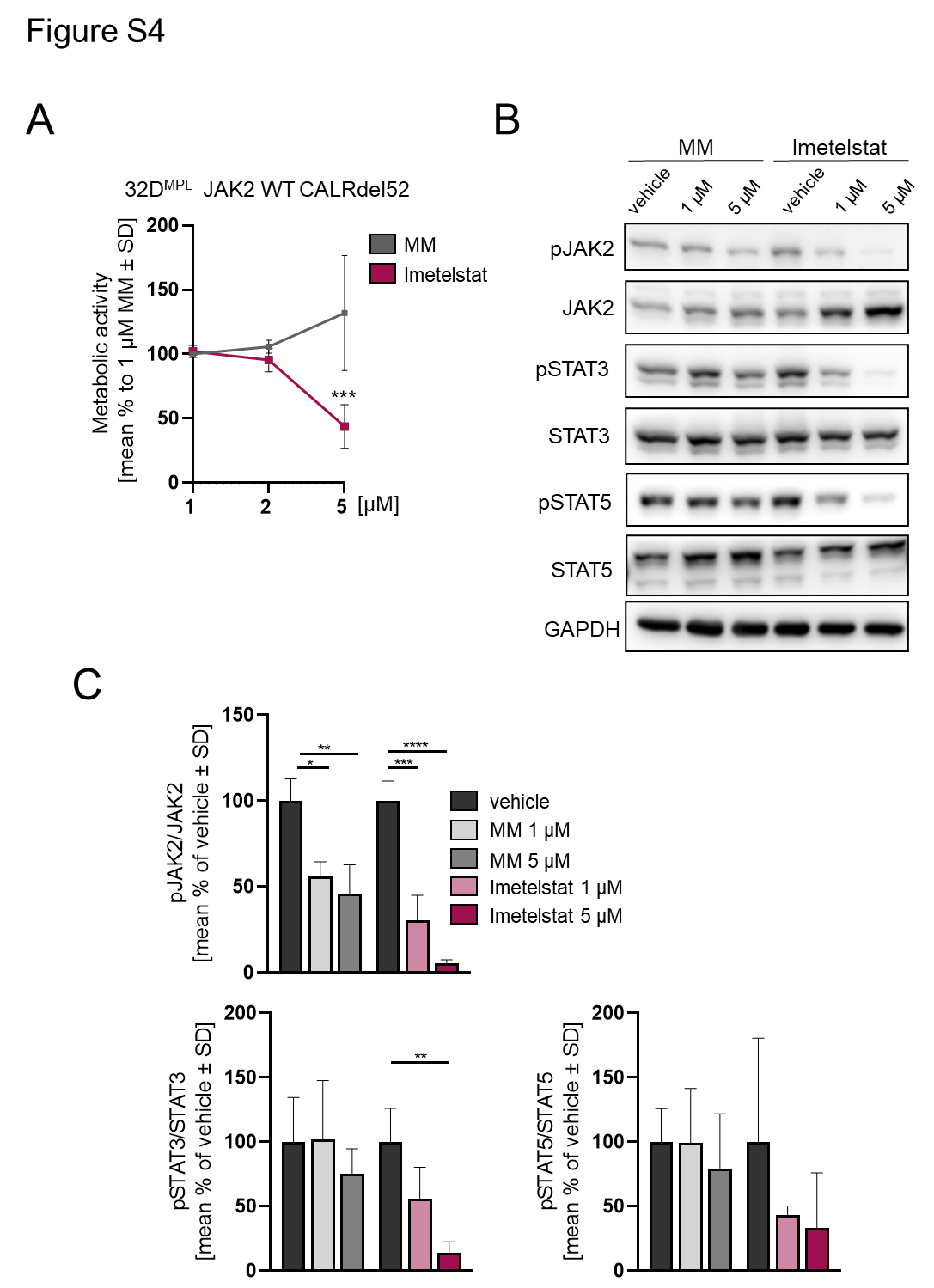


**Figure S4.** (A) MTT assay of 32D^MPL^ cells expressing *JAK2* wildtype and CALRdel52 treated with 1, 2 or 5 µM MM or imetelstat for 72 h. *n*=3. Each cell mean was compared to the other cell mean of the row in a Two-Way ANOVA multiple comparison test (Bonferroni). ****p*<0.001 (B) Western blotting of 32D^MPL^ cells expressing JAK2 wildtype and CALRdel52 after vehicle, MM control or imetelstat stimulation for 24 h h. GAPDH was used as loading control. MM: mismatch control; SD: standard deviation. (C) Densitometric analysis of Western blots of 32D^MPL^ JAK2 and CALRdel52 expressing cells treated with 1, 2 and 5 µM MM or imetelstat for 24 h shown in Figure 3E and 2 more biological replicates. The band intensities of the phosphorylated proteins were normalized to their respective whole proteins. *n*=3. T-test was applied. **p*<0.05, ***p*<0.01, ****p*<0.001, *****p*<0.0001. MM: mismatch control; SD: standard deviation**.**

Table S1. Antibody list for flow cytometry

| **Antibody** | **Company** |
| --- | --- |
| APC mouse anti-human CD34 | BD, USA |
| PE mouse anti-human CD14 clone MEM-18 | Immunotools, Germany |
| APC mouse anti-human CD16 clone LNK16 | Immunotools, Germany |
| FITC anti-human CD61 clone VI-PL2 | BioLegend, USA |
| CD45-APC-Vio770 human | Miltenyi Biotec, Germany |
| CD66b-PE human | Miltenyi Biotec, Germany |
| PE/Cyanina7 anti-human CD41 | BioLegend, USA |
| CD45-APC anti-human | Beckman Coulter. USA |

Table S2. Patient´s clinical course.

| **Days** | **Months** | **Years** | **ASXL1 T880fs_c.2638dupA (%)** | **ASXL1 R965*_c.2893C>T (%)** | **JAK2 V617F_c.1849G>T (%)** | **KRAS T58I _c.173C>T (%)** | **TET2 E1470fs _c.4408delG (%)** | **U2AF1 Q157R_c.470A>G (%)** | **Treatment** | **WBC (/nl)** | **Hgb (g/dl)** | **Hct (%)** | **Plt (/nl)** | **LDH (U/L)** | **Blasts in PB (%)** | **Spleen length (cm below LCM)** |
| --- | --- | --- | --- | --- | --- | --- | --- | --- | --- | --- | --- | --- | --- | --- | --- | --- |
| 0 | 0.00 | 0.00 |  |  |  |  |  |  | HU | 9.5 | 13 | 39 | 357 | 249 |  | 3 |
| 35 | 2.92 | 0.10 | 32 | 8.8 | 50 | 0 | 42 | 42 | HU | 8.6 | 13.4 | 41.1 | 344 | 263 |  | 6 |
| 49 | 4.08 | 0.14 |  |  |  |  |  |  | W&W | 9.7 | 13.8 | 42.2 | 328 | 261 |  | 6 |
| 56 | 4.67 | 0.16 |  |  |  |  |  |  | RUX (15mg BID)-containing regimen | 9.4 | 14.2 | 32.6 | 429 | 283 |  | 6 |
| 68 | 5.67 | 0.19 |  |  |  |  |  |  | RUX (15mg BID)-containing regimen | 9.8 | 13.4 | 39.8 | 396 | 316 |  | 5 |
| 75 | 6.25 | 0.21 |  |  |  |  |  |  | RUX (15mg BID)-containing regimen | 7.6 | 13.4 | 40.5 | 359 | 342 |  | 4 |
| 82 | 6.83 | 0.23 |  |  |  |  |  |  | RUX (15mg BID)-containing regimen | 8.4 | 12.9 | 37 | 297 | 265 |  | 2 |
| 96 | 8.00 | 0.27 |  |  |  |  |  |  | RUX (15mg BID)-containing regimen | 12 | 12.6 | 37.5 | 439 | 410 |  | 1 |
| 124 | 10.33 | 0.34 |  |  |  |  |  |  | RUX (15mg BID)-containing regimen | 11.4 | 12 | 34.9 | 379 | 465 |  | 1 |
| 137 | 11.42 | 0.38 |  |  |  |  |  |  | RUX (15mg BID) | 11.7 | 11.4 | 34.9 | 280 | 468 |  | 0 |
| 151 | 12.58 | 0.42 |  |  |  |  |  |  | RUX (15mg BID)-containing regimen | 12.9 | 10.4 | 32.3 | 377 | 486 |  | 0 |
| 158 | 13.17 | 0.44 |  |  |  |  |  |  | RUX (15mg BID)-containing regimen | 13.7 | 10.6 | 32 | 400 | 551 |  | 0 |
| 167 | 13.92 | 0.46 |  |  |  |  |  |  | RUX (15mg BID)-containing regimen | 10.9 | 10.2 | 30.7 | 344 | 462 |  | 0 |
| 191 | 15.92 | 0.53 |  |  |  |  |  |  | RUX (15mg BID)-containing regimen | 14.4 | 10.4 | 32.5 | 333 | 525 |  | 0 |
| 219 | 18.25 | 0.61 |  |  |  |  |  |  | RUX (15mg BID)-containing regimen | 14 | 11 | 33.7 | 264 | 614 |  | 0 |
| 249 | 20.75 | 0.69 |  |  |  |  |  |  | RUX (15mg BID)-containing regimen | 14.8 | 11.2 | 34.3 | 240 | 601 |  | 0 |
| 276 | 23.00 | 0.77 |  |  |  |  |  |  | RUX (15mg BID)-containing regimen | 24.9 | 11.7 | 35.9 | 312 | 722 | 2 | 0 |
| 332 | 27.67 | 0.92 |  |  |  |  |  |  | RUX (15mg BID)-containing regimen | 25.5 | 12 | 37.2 | 257 | 665 | 1 | 0 |
| 380 | 31.67 | 1.06 |  |  |  |  |  |  | RUX (15mg BID) | 25.8 | 12.6 | 39.5 | 319 | 746 | 2 | 0 |
| 414 | 34.50 | 1.15 |  |  |  |  |  |  | RUX (20mg BID) | 33.4 | 12.6 | 38.2 | 300 | 750 | 4 | 3 |
| 427 | 35.58 | 1.19 |  |  |  |  |  |  | RUX (20mg BID) | 30.4 | 12 | 37.4 | 232 | 693 |  | 3 |
| 489 | 40.75 | 1.36 |  |  |  |  |  |  | RUX (20mg BID) | 30.3 | 12 | 37.2 | 277 | 740 |  | 5 |
| 568 | 47.33 | 1.58 |  |  |  |  |  |  | RUX (25mg BID) | 39.5 | 12.5 | 38.9 | 289 | 723 | 2 | 8 |
| 629 | 52.42 | 1.75 | 36 | 9.1 | 57 | 2.53 | 46 | 45 | RUX (25mg BID) | 33.9 | 11.8 | 37.5 | 198 | 714 | 2 | 4 |
| 711 | 59.25 | 1.98 |  |  |  |  |  |  | RUX (25mg BID) | 42.4 | 12.1 | 37.2 | 226 | 724 | 1 | 4 |
| 800 | 66.67 | 2.22 |  |  |  |  |  |  | RUX (25mg BID) + HU 500mg | 45.7 | 11.4 | 35 | 199 | 606 | 5 | 5 |
| 855 | 71.25 | 2.38 |  |  |  |  |  |  | RUX (20mg BID) + HU (500mg QD) | 55.2 | 10.2 | 32.3 | 95 | 602 | 2 |  |
| 862 | 71.83 | 2.39 |  |  |  |  |  |  | RUX (15mg BID) + HU (500mg QD) | 58.1 | 10.3 | 31.1 | 133 | 602 |  |  |
| 890 | 74.17 | 2.47 |  |  |  |  |  |  | HU (500mg BID) | 56.9 | 10.7 | 34.6 | 145 | 656 | 1 | 11 |
| 904 | 75.33 | 2.51 |  |  |  |  |  |  | W&W | 48.3 | 10.7 | 35.7 | 87 | 561 | 0 |  |
| 918 | 76.50 | 2.55 |  |  |  |  |  |  | Imetelstat  (9.4 mg/kg) | 83.2 | 11.9 | 39 | 129 | 635 | 1 | 11 |
| 1007 | 83.92 | 2.80 |  |  |  |  |  |  | Imetelstat  (9.4 mg/kg) | 6.9 | 11.1 | 36 | 64 | 226 | 0 | 10 |
| 1028 | 85.67 | 2.86 |  |  |  |  |  |  | Imetelstat  (9.4 mg/kg) | 4.8 | 11.5 | 36.6 | 63 | 184 | 0 | 10 |
| 1056 | 88.00 | 2.93 |  |  |  |  |  |  | Imetelstat  (9.4 mg/kg) | 8.7 | 12.8 | 40.6 | 79 | 197 | 0 | 9 |
| 1117 | 93.08 | 3.10 |  |  |  |  |  |  | Imetelstat  (9.4 mg/kg) | 5.1 | 11.6 | 38 | 59 | 339 | 0 | 11 |
| 1137 | 94.75 | 3.16 |  |  |  |  |  |  | Imetelstat  (9.4 mg/kg) | 7.2 | 12.2 | 39.3 | 61 | 190 | 0 | 8 |
| 1145 | 95.42 | 3.18 |  |  |  |  |  |  | Imetelstat  (9.4 mg/kg) | 6.8 | 11.9 | 39.4 | 64 | 168 | 0 | 9 |
| 1151 | 95.92 | 3.20 |  |  |  |  |  |  | Imetelstat  (7.5 mg/kg) | 8.9 | 12.6 | 40.8 | 79 | 159 | 0 | 11 |
| 1158 | 96.50 | 3.22 |  |  |  |  |  |  | W&W | 9.4 | 12.7 | 40.6 | 86 | 173 | 0 | 8 |
| 1179 | 98.25 | 3.28 |  |  |  |  |  |  | Imetelstat  (7.5 mg/kg) | 16.9 | 13.1 | 41.6 | 93 | 196 | 0 | 8.5 |
| 1200 | 100.00 | 3.33 |  |  |  |  |  |  | Imetelstat  (7.5 mg/kg) | 13.2 | 12.9 | 41.4 | 64 | 186 | 0 | 12 |
| 1227 | 102.25 | 3.41 |  |  |  |  |  |  | Imetelstat  (7.5 mg/kg) | 15.8 | 13.2 | 41.6 | 84 | 208 | 0 | 15 |
| 1248 | 104.00 | 3.47 |  |  |  |  |  |  | Imetelstat  (7.5 mg/kg) | 22.5 | 13.5 | 42.1 | 91 | 220 | 0 | 15 |
| 1268 | 105.67 | 3.52 |  |  |  |  |  |  | Imetelstat  (7.5 mg/kg) | 24.1 | 13.3 | 41.9 | 90 | 206 |  | 12 |
| 1289 | 107.42 | 3.58 |  |  |  |  |  |  | W&W | 28.3 | 12.4 | 40.3 | 98 | 278 | 0 | 16 |
| 1309 | 109.08 | 3.64 |  |  |  |  |  |  | Imetelstat  (7.5 mg/kg) | 29.5 | 12.9 | 41.5 |  | 320 | 0 | 16 |
| 1374 | 114.50 | 3.82 |  |  |  |  |  |  | Imetelstat  (7.5 mg/kg) | 21.2 | 13.2 | 43.7 | 102 | 247 | 0 | 13 |
| 1394 | 116.17 | 3.87 | 45 | 0 | 48 | 52 | 44 | 45 | Imetelstat  (7.5 mg/kg) | 24.3 | 13.1 | 41.8 | 108 | 233 | 0 | 13 |
| 1448 | 120.67 | 4.02 |  |  |  |  |  |  | Imetelstat  (7.5 mg/kg) |  |  |  |  |  | 1 | 12 |
| 1539 | 128.25 | 4.28 |  |  |  |  |  |  | Imetelstat  (7.5 mg/kg) | 34.2 | 13.1 | 42 | 139 | 236 | 0 | 13 |
| 1628 | 135.67 | 4.52 |  |  |  |  |  |  | Imetelstat  (7.5 mg/kg) | 37 | 12.5 | 40.3 | 193 | 275 | 0 | 15 |
| 1733 | 144.42 | 4.81 |  |  |  |  |  |  | Imetelstat  (7.5 mg/kg) | 56 | 13.3 | 43.1 | 302 | 377 | 0 | 14 |
| 1754 | 146.17 | 4.87 |  |  |  |  |  |  | Imetelstat  (7.5 mg/kg) | 57.5 | 12.5 | 39.4 | 189 | 439 | 0 | 18 |
| 1760 | 146.67 | 4.89 |  |  |  |  |  |  | RUX (15mgBID) + HU (500mg TID) | 67.1 | 11.8 | 37.1 | 205 | 364 |  | 18 |
| 1822 | 151.83 | 5.06 |  |  |  |  |  |  | RUX (15mgBID) + HU (500mg TID) | 18.8 | 10.6 | 34.4 | 33 | 327 | 0 | 13 |
| 1869 | 155.75 | 5.19 |  |  |  |  |  |  | RUX (15mgBID) + HU (500mg BID) | 28.3 | 11.8 | 37.3 | 54 | 321 | 0 | 12 |
| 1993 | 166.08 | 5.54 |  |  |  |  |  |  | RUX (20mg 0-15mg) + HU (500mg BID) | 70.2 | 11.7 | 38 | 101 | 479 | 0 | 14 |
| 2091 | 174.25 | 5.81 |  |  |  |  |  |  | RUX (20mgBID) + HU (500mg BID) | 67 | 10.7 | 35.5 | 78 | 457 | 1 | 14 |
| 2133 | 177.75 | 5.93 | 48 | 0 | 53 | 54 | 48 | 49 | RUX (20mgBID) + HU (500mg BID) | 85.8 | 11.5 | 36.9 | 53 | 461 | 3 | 14 |
| 2160 | 180.00 | 6.00 |  |  |  |  |  |  | HU (500mg BID) | 58.6 | 10.5 | 34 | 41 | 487 | 2 |  |
| 2182 | 181.83 | 6.06 |  |  |  |  |  |  | HU (500mg BID) | 49.7 | 7.9 | 25.3 | 111 | 424 |  |  |
| 2203 | 183.58 | 6.12 |  |  |  |  |  |  | RUX (10mgBID) + HU (500mg BID) |  |  |  |  |  |  | 18 |
| 2304 | 192.00 | 6.40 | 48 | 0 | 55 | 62 | 47 | 47 | HU (500mg TID) | 34.7 | 10.1 | 35.7 | 44 | 401 | 1 | 18 |
| 2352 | 196.00 | 6.53 |  |  |  |  |  |  | HU (500mg BID) | 25.1 | 9.6 | 33.7 | 36 | 254 |  | 18 |
| 2393 | 199.42 | 6.65 |  |  |  |  |  |  | HU (500mg BID) | 57.7 | 10.7 | 36.2 | 47 | 302 | 2 | 18 |
| 2430 | 202.50 | 6.75 |  |  |  |  |  |  | W&W | 1.9 | 6.2 | 19.3 | 8 | 126 | 4 | 13 |
| 2438 | 203.17 | 6.77 |  |  |  |  |  |  | HU (1000mg BID) | 8.2 | 8.1 | 25.8 | 13 | 145 |  |  |
| 2498 | 208.17 | 6.94 |  |  |  |  |  |  |  | 205.4 | 9.1 | 29.4 | 21 | 473 | 53 |  |

Time is shown as time (days, months, years) since first presentation of the patient at our institution. HU – Hydroxyurea, RUX – Ruxolitinib, W&W – watch and wait, BID – twice a day, TID – three times a day, LCM – Left Costal Margin
